# Sex-Specific Aspects in the Pathophysiology and Imaging of Coronary Macro- and Microvascular Disease

**DOI:** 10.1007/s12265-019-09906-0

**Published:** 2019-08-30

**Authors:** Floor Groepenhoff, Sophie H. Bots, Elise L. Kessler, Ariane A. Sickinghe, Anouk L. M. Eikendal, Tim Leiner, Hester M. den Ruijter

**Affiliations:** 1 Laboratory for Clinical Chemistry and Haematology, University Medical Center Utrecht, Utrecht University, Utrecht, The Netherlands; 2 Laboratory for Experimental Cardiology, University Medical Center Utrecht, Utrecht University, Utrecht, The Netherlands; 3 Department of Radiology, University Medical Center Utrecht, Utrecht University, P. O. Box 85500, 3508 GA Utrecht, The Netherlands

**Keywords:** Coronary artery disease, Microvascular disease, Microvascular dysfunction, Coronary imaging, Sex differences, Fractional flow reserve, Index of microcirculatory resistance, Coronary flow reserve (CFR)

## Abstract

Sex differences in coronary artery disease (CAD) are well established, with women presenting with non-obstructive CAD more often than men do. However, recent evidence has identified coronary microvascular dysfunction as the underlying cause for cardiac complaints, yet sex-specific prevalence numbers are inconclusive. This review summarises known sex-specific aspects in the pathophysiology of both macro- and microvascular dysfunction and identifies currently existing knowledge gaps. In addition, this review describes current diagnostic approaches and whether these should take underlying sex differences into account by, for example, using different techniques or cut-off values for women and men. Future research into both innovation of imaging techniques and perfusion-related sex differences is needed to fill evidence gaps and enable the implementation of the available knowledge in daily clinical practice.

## Introduction

Women suffering from coronary artery disease (CAD), one of the leading causes of death globally, have a worse short- and long-term prognosis than men [1, 2]. They also more often present with clean epicardial arteries (non-obstructive CAD) than men [1], suggesting sex differences are present in the underlying aetiology. The Women’s Ischemia Syndrome Evaluation study found that women with persistent angina complaints and non-obstructive CAD had twice the risk of cardiovascular events compared with women without complaints [3]. Additionally, myocardial perfusion was impaired in approximately half of the women, suggesting that coronary microvascular dysfunction (CMD) plays an important role in the pathophysiology of this condition [4]. Since endothelium-dependent dysfunction can result in decreased perfusion of the myocardium, research suggests that this may be involved in the development of CMD [5].

However, CMD remains difficult to diagnose because the cardiac microvasculature is too small to visualise with conventional imaging techniques. In addition, generalised recommendations regarding treatment for non-obstructive CAD are still lacking [6]. The most recent guidelines from the European Society of Cardiology acknowledge that women with non-obstructive CAD are a special group in need of additional research [7]. This review summarises both the known sex differences in the pathophysiology of (non-)obstructive CAD and the currently available imaging tools for diagnosis, while also identifying evidence gaps and providing some future perspectives.

## Structural and Functional Alterations in Macrovascular Disease

Coronary macrovascular disease or obstructive CAD occurs due to formation and/or rupture of atherosclerotic plaques. Sex differences in atherosclerosis can be observed at different levels. At the risk factor level, diabetes and smoking have a disproportionally large effect on atherosclerosis risk in women compared with men [8]. Other classical risk factors such as hypertension and dyslipidaemia affect the risk equally in both sexes [1].

At the structural level, animal data show that female rodents develop less extensive atherosclerosis under high-fat conditions than male rodents, possibly due to the effect of oestrogens [8]. Activation of oestrogen receptors in female rats suppressed the proliferation of smooth muscle cells (SMCs), an effect that was not seen in males despite the presence of oestrogen receptors on SMCs of both sexes [9, 10]. This suggests that oestrogens may limit the degree of structural alterations in the vasculature in a sex-specific manner. In addition, women more often present with plaque erosion while men more often have plaques prone to rupture [11–13]. An extensive review of the (sex-specific) pathophysiology of atherosclerotic plaque formation is beyond the scope of this review and can be found elsewhere [8, 14].

At the functional level, atherosclerosis-related changes in the vascular wall may reduce arterial compliance, which can lead to hypertension [15]. Hypertension is the most prevalent risk factor for cardiovascular diseases [16] and is more common in elderly women than men [17]. Women with hypertension maintain better systolic function than men but exhibit more stiffening of both the myocardium and the vasculature, suggesting sex differences underlying mechanisms of pressure overload [17].

Clinical observations show that women are more likely to have a normal coronary angiogram (CAG) than men, both when presenting with chest pain complaints [18] or when having a confirmed diagnosis of myocardial infarction (MI) [19]. Data from a sudden coronary death registry showed that 40% of the women who died from CAD did not have any thrombi while this occurred in only 28% of men [11]. This apparent paradox between unobstructed epicardial arteries and poor prognosis most often seen in women may be explained by the presence of microvascular disease.

## Structural and Functional Alterations in Microvascular Disease

The coronary microvasculature modulates the vascular tone through vasoconstriction and vasodilation, which is regulated by systemic and local factors acting on endothelial cells (ECs) and SMCs [20]. The dysregulation of this adaptive system due to structural and functional alterations in the microvasculature is referred to as CMD [21, 22]. Classical macrovascular disease risk factors such as smoking, age, and hypertension may also be associated with impaired microvascular function [23]. Hypertension may disproportionally increase the CMD risk in women because they have lower microvascular arterial compliance than men [24]. Sex differences in structural and functional alterations in CMD have been reported but require validation.

At the structural level, inflammation can affect the microvasculature by inducing SMC proliferation and differentiation of fibroblasts into myofibroblasts [25, 26]. In general, nitric oxide (NO) is needed to maintain the normal functioning and structure of the arteries. NO is produced by endothelial NO synthase (eNOS) in reaction to shear stress of the artery walls, and a drop in NO levels leads to increased perivascular fibrosis and subsequent microvascular stiffening. Such a drop may occur in situations of pressure overload, when cardiomyocyte mitochondria produce free reactive oxygen species (ROS) in response to stress [27, 28]. These free ROS induce endothelial inflammation, which can cause perivascular fibrosis [29]. Oestrogens promote the production of NO and may therefore protect women against structural changes in the microvasculature. However, this possible protective effect of oestrogens is lost with the lack of oestrogens after menopause, possibly leading to increased perivascular fibrosis and subsequent microvascular stiffening. Oestrogens also inhibit collagen I and III deposition through activation of oestrogen receptor (ER) α [30], while androgens such as testosterone promote the deposition of collagen via increasing TGF-β production [31]. This may also contribute to increased perivascular fibrosis in women after menopause.

Capillary dysfunction leads to impaired angiogenesis [32], a process regulated by several myocardium-derived growth factors stimulating endothelial cell growth [33–36]. ERs can act as transcription factors for one of these molecules, vascular endothelial growth factor (VEGF). Animal data show that female mice have a better angiogenic capacity following ischaemia than male mice, suggesting that oestrogens help in limiting myocardial damage after reduced blood flow in female animals [37, 38], possibly via the stimulation of NO production.

Microvascular instability and dysfunction will eventually lead to pruning of vessels, called rarefaction. This decreases the myocardial capillary density, leading to reduced perfusion of the heart and possibly myocardial hypoxia. Reduced myocardial perfusion has been shown to be a major contributing factor in heart failure with preserved ejection fraction (HFpEF) in both women and men [24]. The prevalence and pathophysiology of capillary rarefaction in the heart have not yet been firmly established as autopsy studies in HFpEF patients are rare. The contribution of microvascular rarefaction to CMD and possibly HFpEF in both sexes needs further investigation and might provide an interesting target for therapy in this HF subtype [39].

At the functional level, imbalance between vasodilating and vasoconstricting factors can lead to an impaired vessel response upon changes in oxygen demand and subsequent perfusion defects. The oestrogen receptors ER-α and ER-β can induce vasodilation by activating eNOS, which may have a protective effect on cardiac function. Low oestrogen levels initiate sustained renin-angiotensin-aldosterone system (RAAS) activation, which promotes ROS production and further decreases NO availability [40]. The lack of oestrogen after menopause may thereby lead to an increase in microvascular dysregulation possibly deteriorating into CMD. This together with the presence of cardiovascular risk factors could render women more vulnerable to macro- and microvascular dysfunction after menopause.

## Functional Assessment of the Coronary Microcirculation

While direct visualisation of microvascular abnormalities is still impossible, measurements of the coronary flow enable indirect assessment of microvascular function. Under normal physiological conditions, the coronary microvasculature can induce reactive hyperaemia in response to short or prolonged myocardial ischaemia. In the presence of endothelium-(in)dependent abnormalities, both the reactive hyperaemia response and the subsequent (re)perfusion of the myocardium are suboptimal. Inducing stress can mimic this maximal hyperaemic response for functional assessment of the microcirculation in the clinical setting. The difference in perfusion between healthy and diseased states can be assessed with several invasive and non-invasive imaging techniques and can inform healthcare professionals about the degree of microvascular disease.

### Quantification of Perfusion

Coronary blood flow can be quantified using several different methods. All quantification methods require the use of either an endothelium-dependent [41, 42] or endothelium-independent [43, 44] vasoactive stimulus to achieve maximal hyperaemia. There is no evidence for sex differences in the effect of these stimuli. However, depending on how coronary perfusion is measured, inherent biological sex differences in coronary blood flow may require sex-specific cut-off values for impaired perfusion. We will discuss three commonly used myocardial perfusion metrics below. A more extensive overview can be found in Table 1.

**Table 1:** Sex differences in currently available parameters to quantify coronary perfusion

Parameter name (abbreviation)	Method used to calculate perfusion	Imaging modalities using this parameter to quantify perfusion	Sex differences
Fractional flow reserve (FFR)	P_d_/P_a_ at maximal hyperaemia	CAG	None reported [45–52]
Index of microcirculatory resistance (IMR)	P_d_/absolute coronary flow at maximal hyperaemia	CAG	None reported [45, 48]
Coronary flow reserve (CFR)	Hyperaemic coronary flow/basal coronary flow	CAG, PET, echocardiography, CMR	Ratio possibly lower in women [48]
Myocardial blood flow (MBF)	Absolute myocardial perfusion in mL/min/g	PET, CMR	Rest and stress MBF higher in women. MBF ratio lower in women [53]
Myocardial perfusion reserve index (MPRI)	Myocardial perfusion in stress/myocardial perfusion in rest	CMR	Not yet reported

*CAG* coronary angiography, *CMR* cardiac magnetic resonance imaging, *P*_*a*_ mean proximal coronary artery pressure (mean aortic pressure), *PET* positron emission tomography, *P*_*d*_ mean distal coronary artery pressure

Fractional flow reserve (FFR) is a surrogate estimate of coronary flow based on coronary pressure used to assess the extent of coronary artery stenosis. It is calculated by dividing the distal coronary artery pressure by the mean aortic pressure after maximal vasodilation. The FFR has no sex-specific cut-off point, with a value of 0.8 or higher indicating normal blood flow in both women and men. The FFR cannot differentiate between CAD and CMD, so additional testing is required to confirm CMD in case of an abnormal FFR [45, 54].

The index of microcirculatory resistance (IMR) is a measure of microcirculatory resistance at maximal hyperaemia calculated by dividing the distal coronary pressure by the absolute coronary flow. The IMR is unaffected by resting haemodynamic parameters or epicardial stenosis and provides a more direct measurement of the coronary microcirculatory function compared with the other metrics discussed here [46, 47]. An IMR ≥ 23–25 U is indicative of increased microcirculatory resistance in both women and men [46].

Coronary flow reserve (CFR) is an estimate of coronary perfusion. It measures the maximal blood flow achieved in both epicardial and microvascular vessels in response to hyperaemic stimulation and is calculated by dividing the coronary flow at maximal vasodilation by the coronary flow at rest. Women have a higher resting flow but similar hyperaemic flow compared to men, leading to a lower CFR value with the same degree of microvascular dysfunction [48]. As of yet, there is no consensus on the cut-off for CFR to denote impaired myocardial perfusion, but the underlying sex difference in haemodynamics supports research into the use of a different value for men and women.

### Invasive Imaging Methods

Invasive imaging measures the coronary blood flow velocity at rest and stress during coronary catheterisation using either intra-coronary Doppler flow or thermodilution [49, 55]. Impaired microvascular function measured by Doppler flow was associated with an increased risk of long-term cardiac mortality in patients with ST-elevation myocardial infarction [49] and Doppler-derived CFR correlated better with the non-invasive gold standard positron emission tomography (PET) than thermodilution-derived CFR [56]. However, with thermodilution the CFR and the IMR can be obtained simultaneously [55]. There are no reported differences in effectiveness of these techniques between women and men, but underlying haemodynamic differences must be taken into account when interpreting the results [48].

### Non-invasive Imaging Methods

There are several non-invasive imaging methods available that differ in their approach and use of radiation. PET is considered the gold standard of non-invasive imaging [57], but exposes patients to ionising radiation. Alternatives are transthoracic Doppler echocardiograph (TTDE), cardiac magnetic resonance (CMR) [58], and possibly cardiac computed tomography angiography (CCTA) [59, 60]. These methods are summarised in Table 1.

Data show that measurements obtained by TTDE correlate well with those obtained by invasive Doppler echocardiography [61–63]. CCTA is currently only used for evaluation of calcification and stenosis in the epicardial vessels, but research groups are working on expanding its application to measuring myocardial perfusion and developing computational techniques that can extract flow and pressure data from CCTA images [59, 60]. CMR can detect both perfusion defects and obstructive CAD more accurately than invasive Doppler echocardiography and single-photon emission computed tomography (SPECT), respectively [58, 64]. It thus offers the potential to diagnose both obstructive CAD and CMD in a single examination [65]. There are no reported sex differences for these imaging methods.

## Discussion

In this review, we summarised currently available evidence on sex differences in the pathophysiology and diagnosis of coronary macro- and microvascular disease. In contrast to obstructive CAD, knowledge about non-obstructive CAD and CMD is still lacking on many levels. At the pathophysiological level, sex differences in structural and functional alterations in CMD have been reported but remain understudied. Meanwhile at the clinical level, consensus on the preferred imaging method and perfusion quantification metric is lacking and the prevalence of CMD remains unclear. In addition, perfusion defects are determined using the same cut-off values for men and women, even though research has shown that sex-specific cut-off values may be more appropriate. This unnecessary extra heterogeneity complicates the identification of true differences between women and men. Clear guideline recommendations on the choice of vasoactive stimulus, imaging medium, and perfusion cut-off values will help to streamline and focus research efforts in this field.

### Future Perspectives for CMD Diagnostics

To gain more insight into the pathophysiology and treatment of patients with non-obstructive CAD, easily accessible and low risk diagnostics are needed to identify patients with CMD. Both non-invasive imaging techniques and blood-based biomarkers may provide future diagnostics for CMD [66].

High reliability and lack of radiation make CMR a promising non-invasive imaging technique for diagnosis of CMD. It is currently not considered a standard diagnostic tool for CMD due to the limited availability of imaging equipment and the lack of agreement regarding acquisition and post-processing. Research efforts aimed at facilitating the use of CMR in standard care are working on creating perfusion measurements without the use of contrast injection [67], creating a fully automated absolute perfusion measurement [68], and building new MRI coils that will reduce MRI scanning times from an hour to 15 min [69]. These improvements will make CMR a more feasible and attractive diagnostic option for CMD in the future. Machine learning algorithms that support the imaging specialist in interpreting imaging results can be implemented to improve the accuracy of the diagnosis while reducing the reading time [70]. Machine learning can also be applied to clinical care data. Algorithms built using data from electronic health records are emerging as a tool to help clinicians translate the substantial amount of available data to a diagnosis and appropriate treatment [71]. It is important to stress the use of a sex-specific approach in validating these algorithms, since they are only as unbiased as the data they are based upon. If women are underrepresented in the datasets used to power these models, the algorithm could perform poorly for women. For example, a facial recognition software based on an unbalanced dataset used classifiers that performed better on male faces than female faces [72]. Therefore, proportionate representation of both women and men, but also of ethnic groups, should be ensured before using a dataset to develop healthcare algorithms [73].

Biomarkers can be used to both improve risk stratification for and diagnosis of patients with CAD, possibly reducing the need for imaging in these patients. Several different markers of vascular inflammation, oxidative stress, and some others have been proposed as possible biomarkers for CMD, but have not been established or validated yet [66].

### Treatment Perspectives and Related Diseases

Treatment for obstructive CAD is well established and includes revascularisation via stenting or coronary bypass grafting. Data show that women are less likely to undergo these interventions than men, even when they have been diagnosed with acute coronary syndrome (ACS) [19] and have an equal or even higher risk profile [74]. This sex difference is also apparent in prescribed medication, as women with ACS were less likely to receive β-blockers and statins than men with similar disease severity [75]. Women taking cardiovascular medications are more likely to experience (serious) adverse drug reactions than men [76], which may explain why physicians may choose not to prescribe these drugs for women. However, sex-specific evidence per separate drug class is still too limited [77] to support not prescribing these drugs for women and thereby denying them the advantages of treatment.

Treatment for CMD has been largely empirical due to the lack of knowledge about the pathophysiology and the difficulty of reliably diagnosing the condition. Currently available options include medications already used to treat obstructive CAD and cardiovascular risk factors, such as low-dose aspirin, statins, and β-blockers [78]. Persistent angina symptoms can be reduced by using a device to narrow the coronary sinus [79]. Optimal treatment of CMD is important, as several studies have shown that impaired perfusion of the heart is related to a poor prognosis independent of the imaging modality used [80–82]. Given that the currently prescribed medications have already been in use for other indications, it is likely that the sex differences described for obstructive CAD treatment also hold true for CMD treatment. However, data on this are still lacking due to the novelty of the research field.

CMD can be the precursor of chronic cardiac conditions such as HFpEF [58], a subtype of HF that is more common in women [83]. The prognosis of HFpEF is poor with approximately half of patients dying within 5 years after diagnosis but effective treatments are still lacking [83]. Better understanding and earlier recognition of subclinical conditions such as CMD are therefore crucial to tackle this syndrome early on. Next to improvement of diagnosis of CMD, more research is needed into possible treatments of CMD, the underlying pathophysiology, and possible disease phenotypes that can identify subgroups at higher or lower risk of developing HFpEF [84].

### Conclusion

Sex differences in CAD have been identified at all levels of the disease, but such detailed information is still missing for CMD. While research has suggested the presences of such differences, for example through the effect of sex hormones, many evidence gaps still exist. Currently available imaging techniques enable clinicians to evaluate CMD, but international consensus on the optimal procedure is missing and underlying sex differences in baseline perfusion are not always taken into account. Innovative strategies to improve current diagnostic techniques such as the incorporation of machine learning approaches will hopefully enable clinicians to screen for CMD in standard care. However, these approaches must consider sex differences in their development to avoid the introduction of biases in the end product.

## Abbreviations

CAD: Coronary artery disease
CAG: Coronary angiography
CCTA: Cardiac computed tomography angiography
CFR: Coronary flow reserve
CMD: Coronary microvascular disease
CMR: Cardiac magnetic resonance imaging
ECs: Endothelial cells
FFR: Fractional flow reserve
HFpEF: Heart failure with preserved ejection fraction
IMR: Index of microcirculatory resistance
MBF(R): Myocardial blood flow (reserve)
MPRI: Myocardial perfusion reserve index
PCI: Percutaneous coronary intervention
PET: Positron emission tomography
SMCs: Smooth muscle cells
